# Diverse electrophysiological demyelinating features in a late-onset glycogen storage disease type IIIa case

**DOI:** 10.1515/med-2025-1172

**Published:** 2025-04-29

**Authors:** Xiajun Zhou, Xingxing Zhong, Mingshi Gao, Dongyue Yue, Kai Qiao, Min Wang, Nan Zhi, Wenwei Cao, Lu Han, Jiahong Lu, Wenhua Zhu, Chongbo Zhao, Yangtai Guan

**Affiliations:** Department of Neurology, Renji Hospital, Shanghai Jiaotong University School of Medicine, Shanghai, 200127, China; Department of Neurology, Northern Jiangsu People’s Hospital Affiliated to Yangzhou University, Jiangsu, 225000, China; Department of Pathology, Huashan Hospital, Fudan University, Shanghai, 200040, China; Department of Neurology, Jing’an District Center Hospital, Shanghai, 200040, China; Department of Neurology, and Huashan Rare Disease Center, Huashan Hospital, Fudan University, National Center for Neurological Disorders (NCND), Shanghai, 200040, China; Department of Neurology, Punan Branch of Renji Hospital, Shanghai Jiaotong University School of Medicine (Punan Hospital in Pudong new district), Renji Hospital, 279 Linyi Rd., Shanghai, 200125, China

**Keywords:** glycogen storage disease type III, glycogen debranching enzyme system, demyelinating diseases, electrophysiology

## Abstract

Glycogen storage disease type IIIa (GSD IIIa) is a rare etiology among patients with adult-onset myopathy, which is typically associated with axonopathy rather than demyelination. We report a genetically and pathologically confirmed case that exhibited prominent electrophysiological hallmarks of demyelination, including prolonged distal motor latency, temporal dispersion, prolonged F-waves, and conduction block. The presence of these diverse demyelinating characteristics in this context, excluding other factors, is infrequently reported, suggesting that glycogen accumulation may influence not only muscles but also potentially the myelin, thereby broadening our comprehension of this rare disease spectrum.

## 1 Introduction

Glycogen storage disease type IIIa (GSD IIIa) is a metabolic disorder characterized by prominent muscle involvement among its various phenotypic manifestations [1]. This deficiency of glycogen debranching enzyme, encoded by the *amylo-1,6-glucosidase, 4-alpha-glucanotransferase* (*AGL*) gene at 1p21.2, affects catalysis of glycogen side chain transfer, hydrolysis, and linearization for phosphorylation, resulting in glycogen accumulation in tissues, and presenting hepatomegaly, hypoglycemia, and myopathy [2,3]. Adult-onset GSD IIIa is relatively uncommon in the Chinese population, and descriptions of its nerve conduction characteristics are scarce. Herein, we present a unique case with histologically and genetically confirmed GSD IIIa, exhibiting prominent electrophysiological demyelination features that are rarely linked to metabolic myopathies.

## 2 Case report

A 60-year-old male, standing at a height of 165 cm (BMI: 19.8 kg/m^2^), presented with a decade-long history of numbness in both lower legs and a 5–6-year history of progressive weakness in both limbs. Initially, he suffered from low back pain accompanied by persist numbness in his lower legs, without weakness. However, over the past 5–6 years, he has experienced a gradual onset of weakness in his left lower limb, which has since spread to right. This has significantly impaired his ability to climb stairs, rise from a squat, and walk comfortably on flat surfaces. Approximately 4–5 years ago, he began to notice weakness in both hands, particularly affecting the left. These symptoms have gradually worsened, leading to atrophy in his hands, making it challenging for him to perform tasks such as buttoning clothes or holding chopsticks. Throughout his illness, the patient did not exhibit any symptoms of myalgia, rash, ptosis, dyspnea, dysphagia, muscular pain, urine discoloration, or numbness. While his mother remains alive and healthy, his father has passed away. He has no siblings and has adopted a son. Unfortunately, detailed medical information from his parents is unavailable. During his physical examination, the Medical Research Council’s grading scale revealed the following scores for various muscle groups: neck flexion – 4; shoulder abduction – 5 (left) and 4 (right); elbow flexion – 5 (left) and 3 (right); wrist flexion – 4 (bilaterally); finger flexion and extension – 3 (bilaterally); and hip, knee flexion, and knee extension – 5 (left) and 4 (right). No tendon reflexes were elicited in his extremities. There was evident atrophy of the hand muscles bilaterally, while the thigh muscles showed mild atrophy. There were no signs of calf muscle pseudohypertrophy or winged scapula. Testing for pinprick and vibration sensation revealed only minor impairment. The liver and spleen were not palpable below the costal margin.

During two hospitalizations separated by 17 days, creatine kinase (CK) levels remained high at 593 and 542 u/L (normal <310 u/L). Concurrently, CK isoenzyme-MB levels were elevated at 18.9 and 18.5 ng/mL (normal <6.3 ng/mL), while aspartate aminotransferase levels were mildly elevated at 44 and 47 u/L (normal <40 u/L). The fasting blood glucose level were mildly reduced, reaching 3.48 mmol/L (normal >3.9 mmol/L). Additionally, the myositis antibody test revealed only positive result for anti-phosphorylase B kinase. Other liver-related markers, including alanine aminotransferase, γ-glutamyl transferase, bilirubin, alkaline phosphatase, lactate dehydrogenase, and albumin, showed no clinically significant abnormalities. Besides, glycosylated hemoglobin, thyroid function, immunofixation electrophoresis, cerebrospinal fluid protein levels, and cell counts all remained within normal limits. The patient’s acid alpha-glucosidase level of 3.2 μmol/L/h fell within the normal range of 1.46–20.34 μmol/L/h.

**Figure 1:**
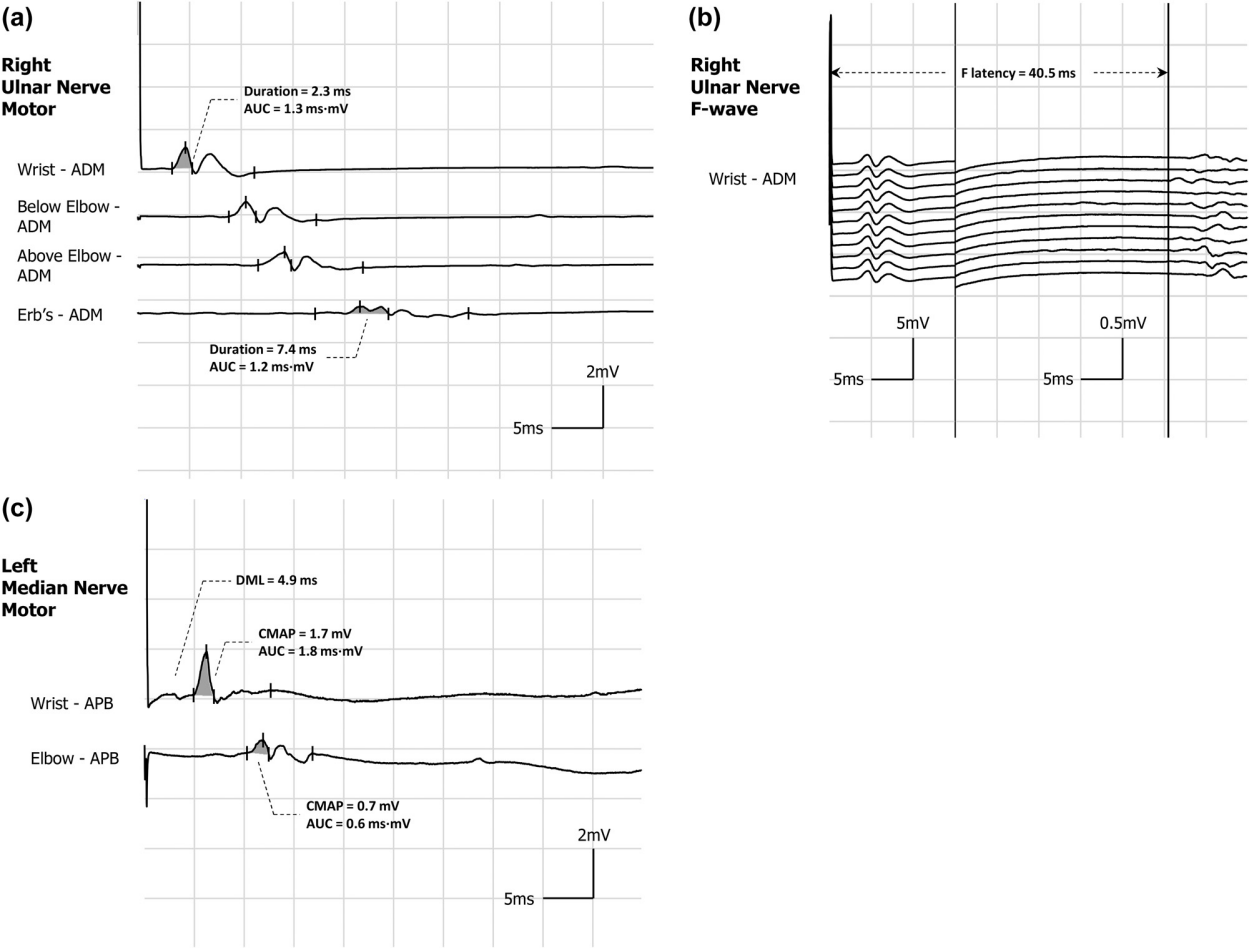
Typical motor nerve conduction waveforms. (a) The left ulnar nerve exhibited a pronounced temporal dispersion. This was characterized by a marked decrease in negative peak amplitude of 65.7% (1.0 vs 0.3 mV) and a total negative peak duration extension of 221.7% (2.3 vs 7.4 ms) when stimulated at Erb’s point compared to distal stimulation, with a non-significant reduction (9.8%) in area (grey shadow). (b) The right ulnar nerve demonstrated a significant prolonged shortest latency of the F wave (40.5 ms). (c) There was evidence of prolongation of distal latency (4.9 ms) accompanied by conduction block in the left median nerve, as indicated by a substantial reduction in proximal stimulus amplitude (0.52 mV) compared to distal stimulation (1.7 mV), resulting in a decrease of 69.4%. Additionally, there was a significant decrease in area (grey shadow) by 62.6%. Additionally, co-stimulation of the ulnar nerve was ruled out by performing distal stimulation of the median nerve while simultaneously recording at the APB and ADM. ADM: abductor digiti minimi; APB: abductor pollicis brevis; AUC: area under curve; CMAP: compound muscle action potential; DML: distal motor latency.

The patient underwent a comprehensive electrophysiological examination (Table 1), revealing characteristic patterns of myogenic impairment unaccompanied by enlargement of motor unit potentials (MUPs). In the motor conduction study, the distal motor latency (DML) of each nerve was prolonged, particularly in the ulnar nerves by exceeding 30%. The conduction velocity of each nerve demonstrated a decrease of about 20–30% compared to distal stimulation, the duration of the left ulnar nerve’s response at Erb’s point was extended by 238% (Figure 1a). Furthermore, the shortest latencies of most F-waves were conspicuously extended, especially in the upper limbs, exceeding 130% (Figure 1b). Proximal stimulation of the left median exhibited a substantial amplitude and area reduction exceeding 60% compared to distal stimulation (Figure 1c). Additionally, there was also a varying decrease in compound motor action potential (CMAP). In the sensory conduction study, a slight decrease in conduction velocity was solely observed in the left median, ulnar, and superficial peroneal nerves.

**Table 1:** Electrophysiological data for this patient

Nerve	Stimulation – record	Latency			Amplitude			Velocity			F-wave		
		ms			mV/μV^*^			m/s			ms		
		Left	Right	LLN	Left	Right	LLN	Left	Right	LLN	Left	Right	ULN
**Motor nerve study**													
Median	Wrist – APB	**4.9↑**	**NP**	4.1	**1.7↓**	**NP**	5.0				**NP**	**NP**	29.0
	Elbow – APB	10.3			**0.5↓**			**38.7↓**	NA	51.0			
Ulnar	Wrist – ADM	**4.5↑**	**3.5↑**	3.1	**1.8↓**	**1.0↓**	5.0				**41.2↑**	**40.5↑**	30.2
	Below elbow – ADM	10.2	8.8		**1.4↓**	**0.7↓**		**37.5↓**	**40.7↓**	50.0			
	Above elbow – ADM	12.9	11.6		**1.3↓**	**0.6↓**		**35.2↓**	**35.2↓**				
	Erb’s – ADM	NA	17.2		NA	**0.3↓**							
Peroneal	Ankle – EDB	**5.2↑**	**5.9↑**	4.9	2.3	2.2	2.0				56.3	**68.3↑**	58.5
	Below fibular head – EDB	12.5	12.7		1.8	1.5		**34.2↓**	**36.0↓**	39.0			
	Above fibular head – EDB	15.7	15.5		1.7	1.5		**29.7↓**	**32.1↓**				
Tibial	Ankle – AH	**6.0↑**	**6.2↑**	5.8	**4.1↓**	**2.8↓**	4.8				**65.8↑**	**62.2↑**	57.0
	Popliteal fossa	15.7	15.6		**1.2↓**	**1.8↓**		**33.0↓**	**33.7↓**	40.0			
**Sensory nerve study**													
Median	Digitis III – wrist	3.4	NA		15.1	NA	6.0	**39.8↓**	NA	49.0			
Ulnar	Digitis V – wrist	3.2	NA		14.1	NA	5.0	**32.7↓**	NA	49.0			
Peroneal superficial	Calf – ankle	2.4	2.5		8.9	10.2	5.0	**38.2↓**	41.6	41.0			
Sural	Calf – ankle	2.4	2.1		23.0	16.5	7.0	47.1	44.2	40.0			

*The amplitude of motor nerve conduction is measured in mV, while the amplitude of sensory nerve conduction is expressed in μV. Values deviating from the reference range are shown in bold. Abbreviations: ADM, abductor digiti minimi; APB, abductor pollicis brevis; AH, abductor hallucis; EDB, extensor digitorum brevis; LLN, lower limit of normal; NA, not available; NP, no potential; ULN, upper limit of normal.

MRI of the thigh muscles demonstrated widespread moderate to severe fatty infiltration and atrophy, with the sartorius, gracilis, and adductor longus muscles being relatively spared (Figure S1). His abdominal ultrasonography revealed calcified foci within the liver, while the positron emission tomography-computed tomography scan solely demonstrated multiple muscle atrophy with normal fluorodeoxyglucose metabolism intake (Figure S2) and intrahepatic bile duct stones. Electrocardiography exhibited ST-T changes and a prolonged Q-T interval, while echocardiography showed left ventricular wall thickening.

**Figure 2:**
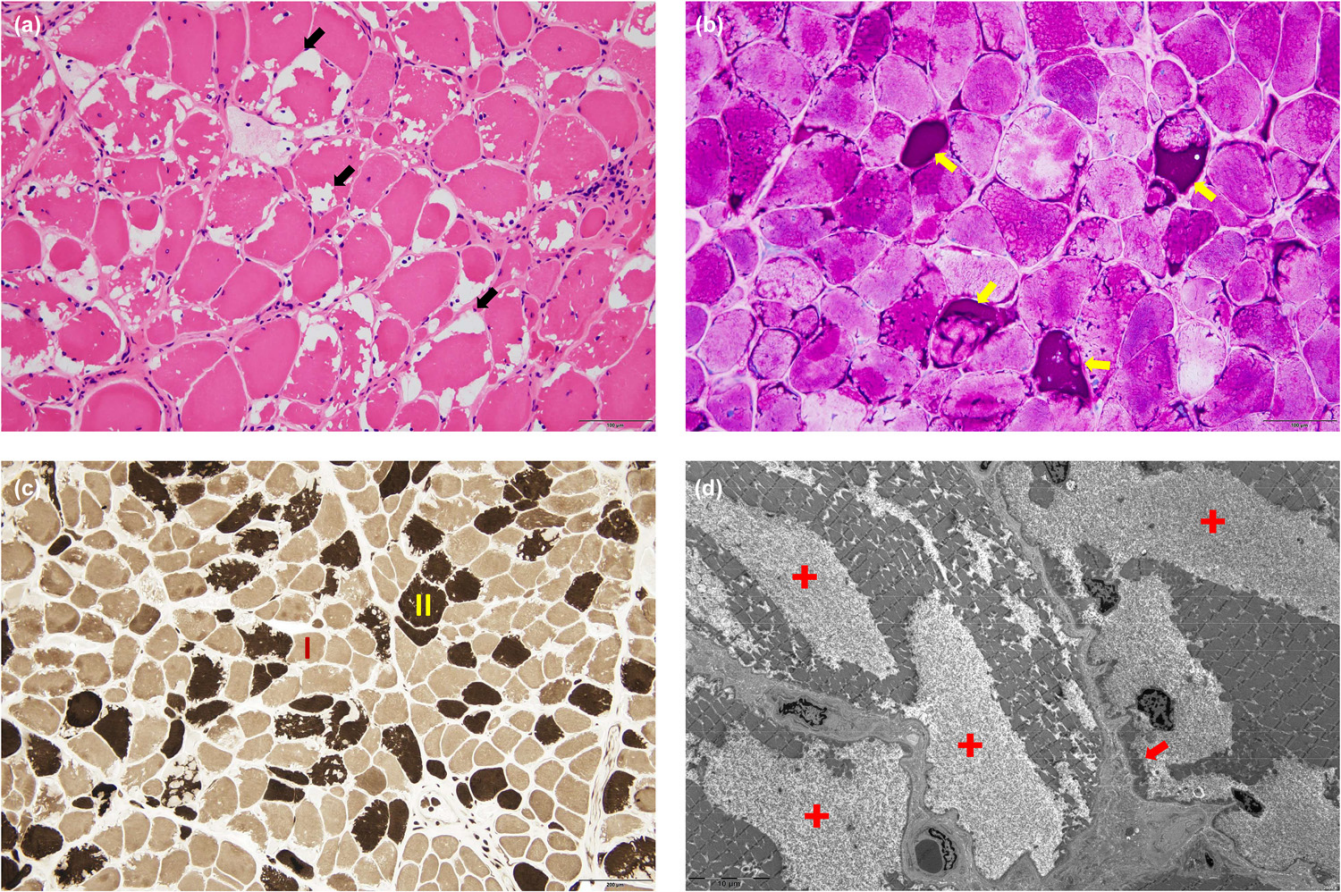
Muscle pathology of the left biceps brachii. (a) HE (200×): marked variation in fiber size, numerous fibers with irregular-shaped sub-sarcolemmal vacuoles (black arrows). (b) PAS (200×): excessive PAS-positive deposits within the vacuoles, indicating the existence of glycogen lakes (yellow arrows). (c) ATPase pH 9.6 (100×): vacuolar fibers involve both type I (light) and type II (dark) muscle fibers. (d) Electron microscopy (2,500×): massive accumulation of glycogen is observed beneath the sarcolemma and between the myofibrils, with most of the glycogen dissolved and glycogen lakes formed (red crosses), while a few glycogen particles (red arrows) remain. ATPase: adenosine triphosphatase; HE: hematoxylin eosin; PAS: periodic acid-Schiff stain.

A muscle biopsy of the left biceps brachii was performed. The hematoxylin and eosin (HE) staining revealed numerous vacuoles of varying sizes beneath the sarcolemma of many muscle fibers, containing uniformly eosinophilic deposits (Figure 2a). Periodic acid-Schiff (PAS) stain further demonstrated strong positivity for deposits within the vacuoles and formation of glycogen lakes (Figure 2b). No other significant changes were observed in enzyme histochemistry staining for modified Gomori trichrome, nicotinamide adenine dinucleotide, cytochrome c oxidase, oil red O, and adenosine triphosphatase (Figure 2c), as well as immunohistochemical staining for dystrophin, sarcoglycan, dysferlin, and desmin. Electron microscopy revealed an extensive increase of dissolved glycogen particles between muscle fibers (Figure 2d).

Whole-exome sequencing analysis uncovered two putative compound heterozygous missense mutations in the *AGL* gene (Figure S3): a known pathogenic deletion-type variant c.2905_2906del (p.Y969Cfs*2) [4], and another novel deletion-type variant c.4479_4481del (p.R1494del).


**Informed consent:** Written informed consent was obtained from the individual for the publication of any potentially identifiable images or data included in this article.
**Ethical approval:** This research involving human subjects has been complied with all the relevant national regulations, institutional policies and in accordance the tenets of the Helsinki Declaration, and has been approved by the independent ethics committee of Renji Hospital Shanghai Jiaotong University.

## 3 Discussion

Given the patient’s age, the significant atrophy observed in the distal upper limbs alongside mild atrophy in the proximal lower limbs, asymmetric weakness, and mild peripheral nerve involvement was evident, the differential diagnosis primarily pointed towards a distal myopathy such as inclusion body myositis (IBM) [5,6]. However, considering the absence of rimmed vacuoles on modified Gomori trichrome staining and the overall histological profile, the likelihood of IBM was discounted, and instead, a metabolism myopathy-like condition was suspected. Previous studies have demonstrated that mutations in genes including *GARS*, *BSCL2*, *REEP1*, and *SH3TC2* underlie distal myopathies primarily affecting the upper limbs, frequently with peripheral nerve involvement [7], and clearly *AGL* should be included in adult-onset distal myopathies, especially when intrinsic hand muscle involvement or IBM-mimicking manifestations are observed. As an adult patient, he currently has no liver-related complaints, nor were any significant morphological liver changes detected on physical examination or imaging. Additionally, apart from a mild elevation in aspartate aminotransferase, his liver function tests remained largely unremarkable across two hospital admissions. The minimal hepatic involvement makes the diagnosis of GSD IIIa more likely than the liver-predominant GSD IIIb subtype.

While metabolic myopathies concurrent with neuropathy have received widespread attention, axonal, including small fiber involvement has been the predominant finding in most reported cases [8,9]. In GSD IIIa, patients have reported subjective sensory disturbances [10,11] and/or slowed sensory conduction velocities [11–13]. In our case, the patient presented with subjective sensory complains along with various electrophysiological manifestations that are consistent with demyelination criteria [14], not merely reduced velocity. These measurements have been repeated to exclude technical factors such as low temperature, insufficient stimulation, and volume conduction. It should be emphasized that the marked decrease in amplitude poses a challenge in unequivocally confirming demyelination. Typically, a decrease in conduction velocity associated with a reduction in motor conduction amplitude indicates either minor secondary demyelination or pseudo-slowing during axon regeneration. However, in cases where the CMAP amplitude falls below 20% of the lower limit of normal values and is supported by demyelination criteria observed in two or more peripheral nerve conductions, the primary consideration should be demyelination [15]. In this case, reductions in motor and sensory velocities, along with other demyelinating features, were observed even when the amplitudes were within or close to the normal ranges, particularly in the peroneal and tibial nerves. Crucially, the absence of further evidence pointing towards axonal degeneration or regeneration, such as enlarged MUP, suggests that the amplitude reduction is solely attributable to myogenic atrophy. Consequently, it appears more plausible to attribute the observed velocity reduction primarily to demyelination, rather than to secondary changes in the axonopathy or other factors.

The patient did not present with any of the typical causes of demyelinating neuropathy, including cerebrospinal fluid albumin-cytologic dissociation, a favorable response to immunosuppressive therapy, or M-proteinemia. Based on the underlying pathophysiological mechanisms, it is rationally conceivable that the occurrence of demyelination in metabolic myopathies is a plausible phenomenon. Previous studies by Ugawa et al. reported a case of GSD IIIa characterized by toluidine blue purplish metachromasia surrounding thin myelin sheaths and PAS-positive reactions in the same fibers. Electron microscopy revealed glycogen deposition in both myelin and axons in this patient [16]. Similarly, another histologically supported report described glycogen deposition in Schwann cells as well as unmyelinated fibers [17]. The deposition of glycogen in neural tissue shows a physical association with amylo-1,6-glucosidase, suggesting that in GSD IIIa, nerve involvement may share similar mechanisms with muscle involvement. These mechanisms may include glycogen accumulation in Schwann cell lysosomes and mitochondrial dysfunction [18], as observed in Pompe disease, which show an increase in lysosomes and autophagosomes in sensory nerves [19,20]. Moreover, studies have indicated that metabolic disorders leading to amino acid imbalance, accumulation of abnormal metabolites (such as 1-deoxy ceramides), and the subsequent inflammation could be potential mechanisms for membrane structural disorder [21]. However, the spatial localization of glycogen and myelin in these diseases, as well as the precise mechanisms of metabolic-inflammation-structural damage, remain unclear. Future challenges may include constructing induced pluripotent stem cell models, precisely regulating *AGL* gene expression through technologies like Perturb-DbiT [22], and using spatial dynamic multi-omics mapping [23] to further explore the interaction between myelin proteins and glycoproteins, as well as the genetic background of myelin susceptibility.

Some variants have been confirmed to be associated with GSD IIIb [1], while others are considered to be linked to the severity of muscle involvement and CK levels [4]. Overall, *AGL* mutations exhibit high genetic heterogeneity, with a weak genotype–phenotype correlation [24] and lack statistical significance [25]. Currently, no genotype has been identified as specifically associated with the neurological phenotype. One of the mutations in this patient is known but has not been reported to be associated with neurological changes [4]; the other novel mutation is located in the putative glycogen-binding domain, and its association with the neurological phenotype requires further validation.

The presented case stands out for its distinct electrophysiological features, i.e., demyelinating dominating over axonal neuropathy, challenging prior assumptions regarding adult-onset GSD IIIa, a metabolic myopathy. It also offers a novel approach for the differential diagnosis of demyelinating peripheral neuropathy. Importantly, when clinicians encounter such diseases with demyelination, they will be more confident in approaching the final diagnosis based on the monism principle, guiding and aiding future treatments targeting both the primary disease and potential myelin repair. A limitation of this study is the inability to obtain myelinated nerve samples. While we believe that morphological validation is of significant scientific value, as an irreversible procedure, we respect the patient’s final decision after a clear diagnosis. Additionally, the specific pathways, mechanisms, and susceptibility related to demyelination remain to be further explored.

## Abbreviations

AGL: amylo-1,6-glucosidase, 4-alpha-glucanotransferase
BMI: body mass index
CK: creatine kinase
CMAP: compound motor action potential
DML: distal motor latency
GSD IIIa: glycogen storage disease type IIIa
HE: hematoxylin and eosin
IBM: inclusion body myositis
MUP: motor unit potential

## Supplementary Material

Supplementary material
