# Supplementary material for: Diverse electrophysiological demyelinating features in a late-onset glycogen storage disease type IIIa case
Source: Open Med (Wars). 2025 Apr 29;20(1):20251172. doi: 10.1515/med-2025-1172 (PMC12048896; doi:10.1515/med-2025-1172)
Supplement: Supplementary material [file med-2025-1172-sm.pdf]

Supplementary material

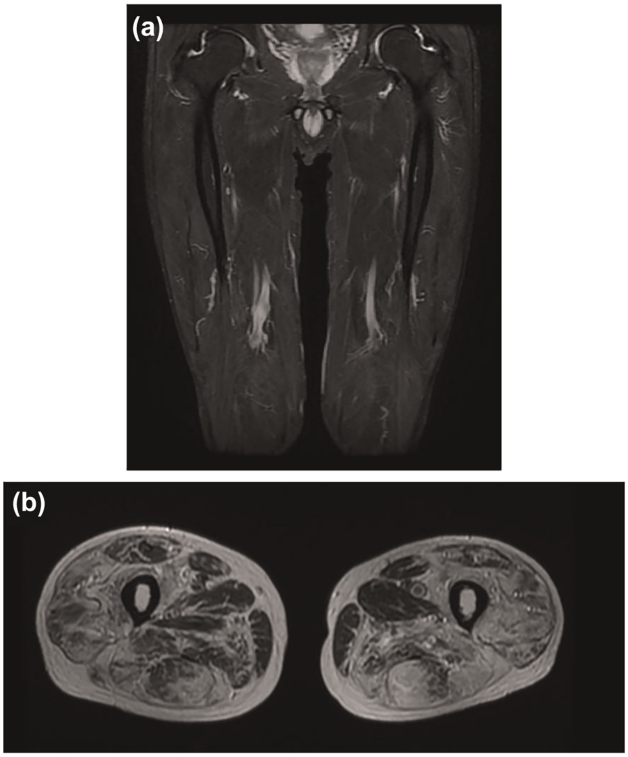

Figure S1: The thigh MRI scan of the patient. (a) STIR and (b) T2 both revealed extensively moderate to severe fatty infiltration and atroph, with the sartorius, gracilis, and adductor longus muscles being relatively spared.

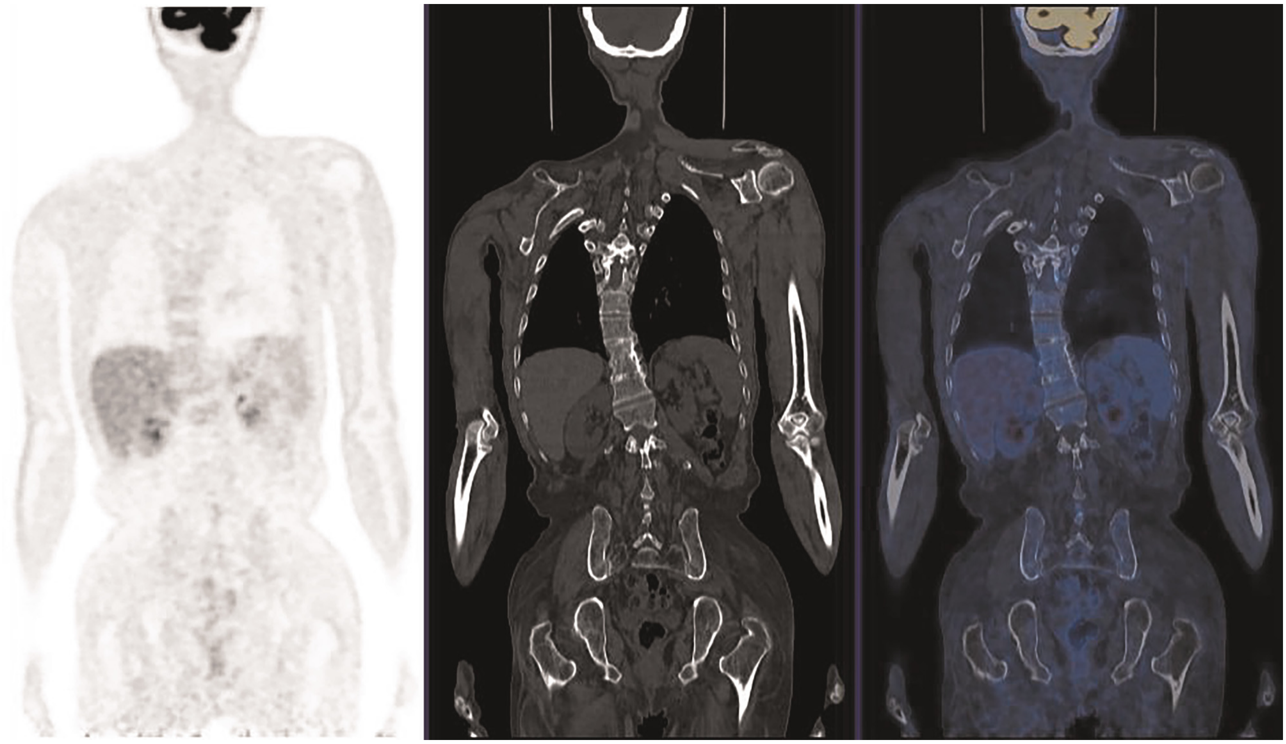

Figure S2: The PET-CT whole-body scan of the patient. It revealed multiple instances of muscle atrophy without any accompanying pathological abnormalities in FDG uptake, ruling out the possibility of inflammatory or neoplastic lesions.

**c.2905_2906del**	
Reference	GATTGACTATGTCAG
Allelic sequence 1	GATTGACTATGTCAG
Allelic sequence 2	GATTGAC---TGTCAG

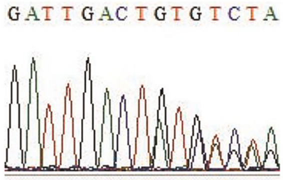

**c.4479_4481del**	
Reference	ATCTTGAGAGGTAAGTCATCAGGAGCATG
Allelic sequence 1	ATCTTGAGAGGTAAGTCATCAGGAGCATG
Allelic sequence 2	ATCTTGA------------------------GAGCATG

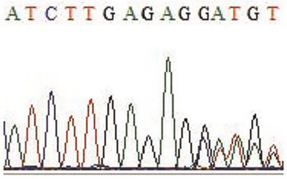

Figure S3: The whole exome sequencing analysis has identified a heterozygous mutation, designated as c2905_2906del (p.Y969Cfs*2), within exon 22 of the AGL gene (transcript NM_000642), specifically located at the chromosomal position chr1-100356868. This mutation site is classified as pathogenic (ACMG: P, PVS1+PM2_Supporting+PM3_Supporting), with prior reports confirming its disease-causing potential. Additionally, a heterozygous mutation, c.4479_4481del (p.R149del), was detected in exon 33 at the chromosomal position chr1-100382285, carrying a variant uncertainty significance (ACMG: VUS, PM2_Supporting+PP4). Based on the clinical and pathological phenotypes observed, it is hypothesized that a compound heterozygous mutation involving both these sites could be pathogenic. Both mutations have been subsequently validated through Sanger sequencing, as illustrated in the accompanying figure.
